# Case Series: Convalescent Plasma Therapy for Patients with COVID-19 and Primary Antibody Deficiency

**DOI:** 10.1007/s10875-021-01193-2

**Published:** 2021-12-10

**Authors:** Julia Lang-Meli, Jonas Fuchs, Philipp Mathé, Hsi-en Ho, Lisa Kern, Lena Jaki, Giuseppe Rusignuolo, Susanne Mertins, Vivien Somogyi, Christoph Neumann-Haefelin, Frederik Trinkmann, Michael Müller, Robert Thimme, Markus Umhau, Isabella Quinti, Dirk Wagner, Marcus Panning, Charlotte Cunningham-Rundles, Katharina Laubner, Klaus Warnatz

**Affiliations:** 1 Department of Medicine II, Medical Center – University of Freiburg and Faculty of Medicine, University Hospital Freiburg, Freiburg, Germany; 2 Institute of Virology, University of Freiburg and Faculty of Medicine, University Hospital Freiburg, Freiburg, Germany; 3 Division of Infectious Diseases, Department of Medicine II, Medical Center – University of Freiburg and Faculty of Medicine, University Hospital Freiburg, Freiburg, Germany; 4 Department of Medicine, Icahn School of Medicine at Mount Sinai, New York, NY USA; 5 Department of Pneumology and Critical Care Medicine, Thoraxklinik, University of Heidelberg, Translational Lung Research Enter Heidelberg, German Center for Lung Research, Heidelberg, Germany; 6 Department of Biomedical Informatics at the Center for Preventive Medicine and Digital Health (CPD-BW), Medical Faculty Mannheim, Heidelberg University, Mannheim, Germany; 7 Institute for Transfusion Medicine and Gene Therapy, University of Freiburg and Faculty of Medicine, University Hospital Freiburg, Freiburg, Germany; 8 Department of Molecular Medicine, Sapienza University of Rome, Rome, Italy; 9 Department of Pediatrics, Icahn School of Medicine at Mount Sinai, New York, NY USA; 10 Department of Rheumatology and Clinical Immunology, Medical Center – University of Freiburg and Faculty of Medicine, University Hospital Freiburg, Breisacher Str. 115, 79106 Freiburg, Germany; 11 Center for Chronic Immunodeficiency, Medical Center – University of Freiburg and Faculty of Medicine, University Hospital Freiburg, Freiburg, Germany

**Keywords:** COVID-19, SARS-CoV-2, Convalescent plasma, Hypogammaglobulinemia, Common variable immunodeficiency, Inborn errors of immunity, Primary immunodeficiencies

## Abstract

Patients with primary antibody deficiency are at risk for severe and in many cases for prolonged COVID-19. Convalescent plasma treatment of immunocompromised individuals could be an option especially in countries with limited access to monoclonal antibody therapies. While studies in immunocompetent COVID19 patients have demonstrated only a limited benefit, evidence for the safety, timing, and effectiveness of this treatment in antibody-deficient patients is lacking. Here, we describe 16 cases with primary antibody deficiency treated with convalescent plasma in four medical centers. In our cohort, treatment was associated with a reduction in viral load and improvement of clinical symptoms, even when applied over a week after onset of infection. There were no relevant side effects besides a short-term fever reaction in one patient. Longitudinal full-genome sequencing revealed the emergence of mutations in the viral genome, potentially conferring an antibody escape in one patient with persistent viral RNA shedding upon plasma treatment. However, he resolved the infection after a second course of plasma treatment. Thus, our data suggest a therapeutic benefit of convalescent plasma treatment in patients with primary antibody deficiency even months after infection. While it appears to be safe, PCR follow-up for SARS-CoV-2 is advisable and early re-treatment might be considered in patients with persistent viral shedding.

**Supplementary Information:**

The online version contains supplementary material available at 10.1007/s10875-021-01193-2.

## Introduction

Known risk factors for severe outcomes of COVID-19 in the general population include age, sex, diabetes mellitus, and underlying cardiovascular disease [1]. While primary immunodeficiency as a whole group did not seem to add by itself to the risk of a severe COVID-19 course, some specific immunodeficiencies were associated with an increased risk [2, 3]. This includes patients with deficiencies of the interferon response either genetically [4, 5] or due to their phenocopies by antibodies against interferon alpha and omega [6, 7] and patients with auto-antibodies neutralizing type I interferons due to autoimmune polyendocrine syndrome type-1 syndrome [8]. Single reports suggest an increased risk of severe COVID19 in patients with NFKB2 deficiency [9] and reports including a large number of patients with common variable immunodeficiency (CVID) suggested a higher fatality rate from SARS-CoV-2 infections among a subgroup [10, 11].

The effective immune response against COVID-19 comprises the innate immune system including an interferon response and the adaptive immune system including an early CD8 T cell response with subsequent CD4 and antibody response [12–15]. Interestingly, a delayed antibody response was associated with a worse outcome [16]. Given the poor humoral immune response in patients with antibody deficiencies, one obvious therapeutic option to treat COVID-19 is convalescent plasma, as its efficacy has been demonstrated for several other viral infections such as SARS-CoV, H5N1, or H1N1 [17–19]. In immunocompetent individuals with mild COVID-19 but high risk for disease progression, administration of convalescent plasma less than 72 h after the onset of symptoms significantly reduced the progression to severe COVID-19 [20]. However, administration of convalescent plasma to hospitalized patients with already-established severe COVID-19 pneumonia did not result in a clinical benefit [21]. Similarly, a recent large randomized controlled trial of convalescent plasma in hospitalized patients with severe disease (receiving oxygen supplementation) up to 12 days after symptomatic onset did not show a therapeutic benefit of convalescent plasma but participants receiving convalescent plasma experienced more adverse events depending on the plasma preparations [22]. Therefore, in immunocompetent individuals, treatment with convalescent plasma seems to be only beneficial in the early phase of infection and caution regarding the selection of plasma donors is warranted. For patients with underlying primary antibody deficiency like CVID, evidence on therapeutic management is restricted to single case reports [23–25]. More case reports and a few case series of patients with secondary immunodeficiency support the idea, that convalescent plasma therapy is beneficial [26–29]. However, single cases with fatal COVID-19 raised concerns that treatment with convalescent plasma during chronic infection may drive viral evolution and result in SARS-CoV-2 variants with a decreased sensitivity to neutralizing antibodies [30–32]. Thus, given the lack of specific antibody responses and the important role of the humoral immune response in the timely control of the infection, treatment with convalescent plasma or monoclonal antibodies against SARS-CoV-2, where it is available, seems to be a rational option, but may require monitoring for viral escape variants. Here, we describe the clinical outcome of 16 patients with primary antibody deficiency from four centers after treatment with convalescent plasma.

## Materials and Methods

### Patient Cohort and Clinical Data

Physicians from four centers (Departments of Medicine and Pediatrics, Mount Sinai School of Medicine, New York, USA; Center for Chronic Immunodeficiency, University of Freiburg, Freiburg, Germany; Department of Molecular Medicine, Sapienza University of Rome, Rome, Italy and Thoraxklinik at the University Hospital Heidelberg, Heidelberg, Germany) were asked to retrospectively complete a questionnaire on their patients with chronic antibody deficiency and documented SARS-CoV-2 infection who received treatment with convalescent plasma. SARS-CoV-2 infection had to be confirmed by qPCR. None of the patients was vaccinated before or during their SARS-CoV-2 infection. The anonymized questionnaire inquired demographic data, COVID-19 presentation, treatment details, and outcomes. The study was approved by the Ethics Committee of the University Hospital of Freiburg (approval number FR 354/19) and patients provided written consent at the respective Centre.

### Classification of COVID-19 Clinical Severity

We assessed clinical severity according to the World Health Organization (WHO) Clinical management Guideline of May 2020 (available at: https://www.who.int/publications/i/item/WHO-2019-nCoV-clinical-2021-1).

### Preparation of Convalescent Plasma

Convalescent plasma donors with a previous SARS-CoV-2 infection were selected and the plasma products were prepared according to local guidelines:

### Mount Sinai School of Medicine, New York, USA

As described before [33], convalescent plasma donors with total anti-spike IgG titers of ≥ 1:320 on the Mount Sinai Hospital-ELISA were referred for plasmapheresis at the New York Blood Center after standard screening for blood donors (e.g., testing for HIV, HAV, HBV, HCV, HEV, PB19). Each unit, approximately 250 ml in volume, was infused over 1–2 h. Convalescent plasma recipients were monitored every 15 min for signs of transfusion-related reactions and then followed for outcomes after the transfusion.

### University Hospital Freiburg, Freiburg and University of Heidelberg, Translational Lung Research Center Heidelberg, Heidelberg, Germany

Convalescent plasma donors above 18 years of age with high anti-spike IgG titres (required ratio > 4, ratio ≥ 1.1: positive; semiquantitative SARS-CoV-2-S1 IgG Euroimmun ELISA calculating a ratio from the extinction of the sample and that of the calibrator) were referred for plasmaphereses at the Institute for Transfusion Medicine and Gene Therapy, University of Freiburg. Standard screening according to the German guideline for hemotherapy was performed (including screening for infectious diseases (HIV, HAV, HBV, HCV, HEV, PB19) via serology and PCR at the time point of donation and 14 days after donation). Donors were accepted at least 42 days after the last positive SARS-CoV-2 PCR and at least 28 days after resolution of symptoms. Before donation, a current negative SARS-CoV-2 PCR from nasopharyngeal swab was required. Convalescent plasma units were infused over 1–2 h. Convalescent plasma recipients were monitored during and after transfusion according to the German guidelines for transfusion surveillance.

### Sapienza University of Rome, Rome, Italy

Patients with documented COVID‐19, completely recovered by at least 14 days and two consecutive negative PCR tests, were considered convalescent plasma donors and screened according to the Italian rules to protect the health of apheresis donors. A positive SARS-CoV-2 serology was required. Convalescent plasma donors were screened for infectious diseases (according to the applying hemotherapy guidelines and additionally for hepatitis A and E viruses and parvovirus B19). The convalescent plasma product was processed with a pathogen reduction method.

### Neutralization

Neutralization experiments with SARS-CoV-2 were performed under Biosafety Level 3 (BSL3) protocols at the Institute of Virology, Freiburg, approved by the Regierungspraesidium Tuebingen (No. 25–27/8973.10–18 and UNI.FRK.05.16–29). To assess the neutralizing capacity of the plasma, serial plasma dilutions were incubated with 100 plaque forming units (pfu) of the prototypic B.1 virus isolate (Muc-IMB-1) for 1 h. The mixture was dispersed on African green monkey kidney VeroE6 cells (ATCC CRL-1586) in 12-well format and the cells were overlaid with 0.6% Oxoid-agar for 48 h at 37 °C. The fixed cells were stained with Crystal violet. Number of plaques was compared with an untreated control without serum.

### Whole Genome Sequencing

cDNA was produced from extracted RNA of oropharyngeal swabs using random hexamer primers and Superscript III (ThermoFisher) followed by a PCR tiling the entire SARS-CoV-2 genome (ARTIC V3 primer sets). The amplicons were cleaned with AMPure magnetic beads (Beckman Coulter). Afterwards, the QIAseq FX DNA Library Kit (Qiagen) was used to prepare indexed paired end libraries for sequencing on a Illumina MiSeq instrument.

### Bioinformatics

The de-multiplexed raw reads were subjected to a custom Galaxy pipeline, which is based on bioinformatics pipelines on usegalaxy.eu [34]. The raw reads were pre-processed with fastp (v.0.20.1) [35] and mapped to the SARS-CoV-2 Wuhan-Hu-1 reference genome (Genbank: NC_045512) using BWA-MEM (v.0.7.17) [36]. Primer sequences were trimmed with ivar trim (v1.9) (https://andersen-lab.github.io/ivar/html/manualpage.html). Variants (SNPs and INDELs) were called with the ultrasensitive variant caller LoFreq (v2.1.5) [37]. Finally, consensus sequences were constructed by bcftools (v.1.1.0) [38]. Regions with low coverage > 20 × or variant frequencies between 30 and 70% were masked with Ns.

### Phylogenetic and Variant Analysis

All available sequences from Germany deposited in GISAID (http://gisaid.org/) between October and November 2020 were downloaded (as of the 22nd of April 2021) and 250 sequences randomly subsampled (Fig. E1). For the phylogenetic analysis, the sequences were first aligned with MAFFT (v7.45) and a tree was constructed with IQ-Tree (v2.1.2). The best fitting substitution model was automatically determined and the tree was calculated with 1000 bootstrap replicates. Branch support was approximated using the Shimodaira–Hasegawa [SH]-aLRT method (1000 replicates). The tree was rooted to the reference sequence NC_045512. The clades were classified with the webservers of Nextclade (clades.nextstrain.org) and Pangolin (pangolin.cog-uk.io). The phylogenetic was visualized with ggtree (v2.2.4) [39], treeio (v1.12.0) [40], and ggplot2 (v3.3.3) packages [41]. An in-house R script was also used to plot the variant frequencies that were detected by LoFreq as a heatmap (github.com/jonas-fuchs/SARS-CoV-2-analyses).

## Results

### Characterization of Patient Cohort

A total of 16 patients (7 female and 9 male; age ranged from 11 to 71 years) with primary antibody deficiency were included in the analysis (Table 1). Most patients were diagnosed with CVID (10/16 patients, one of whom due to NFKB2 deficiency), four patients with X-linked agammaglobulinemia (XLA), one patient with immunodeficiency due to Kabuki syndrome and one with Hyper-IgM syndrome of unknown origin. At the time of SARS-CoV-2 infection, all patients received immunoglobulin substitution as standard therapy for the underlying antibody deficiency and for all but three IgG levels were documented within the therapeutic range. General risk factors for severe COVID-19 which also apply to patients with primary immunodeficiency [10] comprised arterial hypertension in two, cardiac, renal, and chronic obstructive pulmonary disease in one each and chronic interstitial lung disease as part of the underlying immunodeficiency in six patients of our cohort. The latter manifestation had previously been associated with increased COVID-19-related mortality in CVID in the UK [11]. One patient with NFKB2 deficiency was treated because of her progressive disease and of critical COVID-19 previously reported in young patients with this genetic disorder [9, 10].

**Table 1:** Clinical baseline characteristics of the patient cohort with primary antibody deficiency. Risk factors for severe COVID-19 were inquired according to the world health organization clinical management guidance (underlying noncommunicable diseases (NCDs): diabetes, hypertension, cardiac disease, chronic lung disease, cerebrovascular disease, chronic kidney disease, immunosuppression, and cancer; smoking) *CVID* common variable immunodeficiency, *NFKB2* NKFB2 deficiency, *XLA* X-linked agammaglobulinemia

Pat. no	Center	Age	Sex	PID diagnosis	Organ involvement due to primary antibody deficiency	Pre-existent immuno-supressive treatment? (y/n)	IgG level at time of COVID (g/l)	IgA level at time of COVID (g/l)	IgM level at time of COVID (g/l)	Underlying noncommunicable diseases as risk factors for severe COVID-19 disease
1	Freiburg	61	Male	CVID	Splenomegaly, lymphadenopathy, autoimmune cytopenia, renal disease	y; prednisolone 5 mg/d	12,78	< 0,05	< 0,05	Hypertension, chronic kidney disease
2	Freiburg	55	Female	CVID	Interstitial lung disease, airway disease, splenomegaly, lymphadenopathy, autoimmune cytopenia, hepatopathy	y; budesonide 9 mg/d	12,31	< 0,05	< 0,05	Chronic lung disease
3	Freiburg	71	Male	CVID	Airway disease	n	9,26	< 0,05	0,06	Hypertension, cardiac disease
4	Freiburg	30	Female	CVID (NFKB2)	None	n	8,03	< 0,05	0,11	None
5	Freiburg	53	Female	CVID	Interstitial lung disease, airway disease	n	6,58	< 0,05	< 0,05	Chronic lung disease
6	Rome	48	Female	CVID	Interstitial lung disease, airway disease, splenomegaly, lymphadenopathy, autoimmune cytopenia	n	7,20	0,00	0,00	Chronic lung disease
7	New York	66	Female	CVID	None	n	6,66	0,08	0,12	None
8	New York	41	Male	XLA	None	n	10,57	< 0,07	< 0,07	None
9	New York	25	Male	XLA	None	n	8,21	< 0,07	< 0,07	None
10	New York	11	Male	XLA	None	n	NA	< 0,07	< 0,07	None
11	New York	13	Male	XLA	None	n	8,41	< 0,07	< 0,07	None
12	New York	57	Male	Hyper IgM	Interstitial lung disease, splenomegaly, Lymphadenopathy, autoimmune cytopenia	n	9,13	0,07	2,47	Chronic lung disease
13	New York	35	Male	Kabuki	Other autoimmunity, mental disability	n	6,56	0	1,18	None
14	New York	57	Female	CVID	Interstitial lung disease, Lymphoma	n	10,16	< 0,07	< 0,07	Chronic lung disease
15	New York	55	Female	CVID	Interstitial lung disease	n	9,96	< 0,06	0,21	Chronic lung disease
16	Heidelberg	60	Male	CVID	Splenomegaly, lymphadenopathy	n	7,88	< 0,05	2,60	Chronic lung disease

**Fig. 1:**
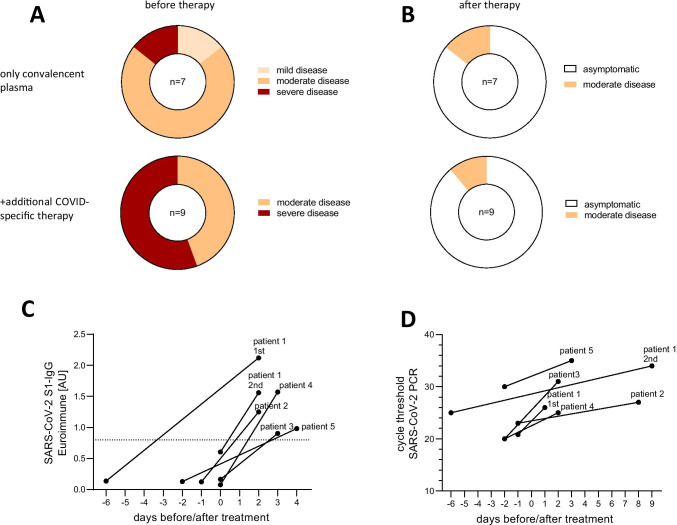
**A** Worst WHO clinical severity score before convalescent plasma therapy. **B** At last follow-up: moderate disease = clinical signs of pneumonia but SpO_2_ ≥ 90% on room air; severe disease = respiratory rate > 30 breaths/min or SpO_2_ < 90% on room air. **C** SARS-CoV-2 IgG titres (Euroimmun ELISA; < 0.8 = negative) and **D** cycle threshold values before/after convalescent plasma treatment. AU = arbitrary units, WHO = World Health Organization

The most common symptoms of COVID-19 in our patient cohort were fever (14/16 patients), cough (11/16 patients), and dyspnoea (10/16 patients). Seven patients received a monotherapy with convalescent plasma, three additionally Remdesivir, one dexamethasone, and five the combination of both (Table 2). Clinical severity of COVID-19 before therapy with convalescent plasma was rated according to the World Health Organization COVID-19 Clinical management guidance [42] (Fig. 1A). One patient presented with mild disease (no evidence for viral pneumonia, but new onset of ataxia). Nine patients were classified with moderate disease (evidence for viral pneumonia, oxygen saturation > 90% on room air) and six patients had severe COVID-19 (viral pneumonia, oxygen saturation < 90% on room air or increased respiratory rate > 30/min). No patient showed critical symptoms (acute respiratory distress syndrome or sepsis). Patients with severe disease were more likely to receive additional COVID-specific therapy.

**Table 2:** Clinical severity level of COVID-19 according to the WHO clinical management guidance: mild disease (no evidence of viral pneumonia), moderate disease (clinical signs of pneumonia like fever, cough, dyspnea, fast breathing, SpO_2_ ≥ 90% on room air), severe disease (plus: respiratory rate > 30 breaths/min or SpO_2_ < 90% on room air), critical disease (acute respiratory distress syndrome or sepsis)

	COVID-19				Convalescent plasma therapy			Additional supportive therapy	Outcome		
Pat. no	COVID-specific symptoms	Worst clinical severity level of COVID-19 before plasma therapy	Pneumonia in imaging (y/n)	Intensive care unit? (y/n)	Time point (days after symptomatic onset)	Side effects? (y/n)	Additional COVID-19-specific therapy?	Antibiotic treatment	Last follow-up (days after symptomatic onset)	Clinical severity level of COVID-19 at last follow-up	First SARS-CoV-2 negative PCR (days after symptomatic onset)
1	Anosmia, fever, cough, fatigue, dyspnoea	Moderate disease	y	n	13, 14 and 62, 63	n	No	None	96	Asymptomatic	96
2	Cough, fever, cephalgia	Moderate disease	y	n	48, 49	n	No	None	83	Asymptomatic	83
3	anosmia, dyspnea	Moderate disease	ND	n	13, 16	y, self-limiting fever	Remdesivir	None	103	Asymptomatic	25
4	Fever, cough, cephalgia	Severe disease	y	n	8, 9	n	Remdesivir, dexamethason	Ampicillin/sulbactam, clarithromycin	65	Asymptomatic	26
5	Fever, cephalgia, loss of appetite, dyspnoea, sore throat	Moderate disease	y	n	132, 133	n	No	Piperacillin/tazobactam, gentamycin per inhalationem	136	Asymptomatic	Unknown
6	Fever, dyspnoea	Severe disease	y	n	61, 63	n	No	Unknown	81	Moderate disease	75
7	Cough, dyspnea, fever, fatigue, weakness	Moderate disease	y	n	34	n	No	Azithromycin	342	Asymptomatic	57
8	Cough, dyspnea, fever, weakness	Moderate disease	y	n	51	n	Dexamethason	Azithromycin	73	Asymptomatic	Unknown
9	Cough, dyspnea, fever, diarrhea	Moderate disease	y	n	27	n	No	Azithromycin	217	Asymptomatic	Unknown
10	Cough, subjective fever, diarrhea, emesis, chest pain	Severe disease	y	n	17	n	Remdesivir	Vancomycin, cefepime, ceftriaxone, azithromycin	188	Asymptomatic	Unknown
11	Cough, fever	Moderate disease	y	n	6	n	Remdesivir	Ceftriaxone, azithromycin	13	Asymptomatic	Unknown
12	Cough, fever, fatigue	Severe disease	y	n	23	n	Remdesivir, dexamethason	Azithromcyin	128	Asymptomatic	Unknown
13	Cough, dyspnea, fever	Severe disease	y	n	8	n	Remdesivir, dexamethason	None	102	Asymptomatic	Unknown
14	Fever, cough, dyspnea, emesis, chills	Moderate disease	y	n	10	n	Remdesivir, dexamethason	None	49	Asymptomatic	Unknown
15	Ataxia	Mild disease	n	n	10, 17	n	No	None	78	Asymptomatic	Unknown
16	Fever, dyspnoea	Severe disease	y	y	5, 6	n	Remdesivir, dexamethason	Ampicillin/sulbactam, clarithromycin, piperacillin/tazobactam, azithromycin	26	Moderate disease	25

### Tolerability and Clinical Response to Treatment with Convalescent Plasma

Convalescent plasma was administered to eight patients within the first 2 weeks after symptomatic onset of COVID-19 because of high risk for disease progression and to the other eight patients between 17 and 132 days because of a long-term SARS-CoV-2 infection (Table 2). Nine patients from New York received a single dose while all others received two doses on subsequent days. One patient required a second treatment course. In our cohort, the treatment was without side effects except for a short febrile period in patient 3. Clinical symptoms improved in all patients after therapy and 15 were asymptomatic at last follow-up (Fig. 1B and Table 2). Five patients with close follow-up reported that the clinical response occurred within 7 days of treatment. Of these five patients, three (patient 2, patient 5, and patient 1 s course) were treated because they showed a long-term SARS-CoV-2 infection.

### Antiviral Response After Treatment with Convalescent Plasma

For five patients from Freiburg (Germany), SARS-CoV-2 serology (SARS-CoV-2 S1 IgG, Euroimmun ELISA) and SARS-CoV-2 qPCR from nasopharyngeal swab were available within 1 week before and after convalescent plasma therapy (Fig. 1C, D). All were seronegative before treatment. The detection of SARS-CoV-2 antibody titers after plasma treatment was associated with an increase of the cycle threshold (ct) values for SARS-CoV-2 qPCR in all patients (Fig. 1C, D). The antiviral effect of the treatment regimen was independent of the time point of treatment with convalescent plasma (8–133 days after symptomatic onset) (Table 2).

### Evidence for SARS-CoV-2 Evolution After Treatment with Convalescent Plasma

**Fig. 2:**
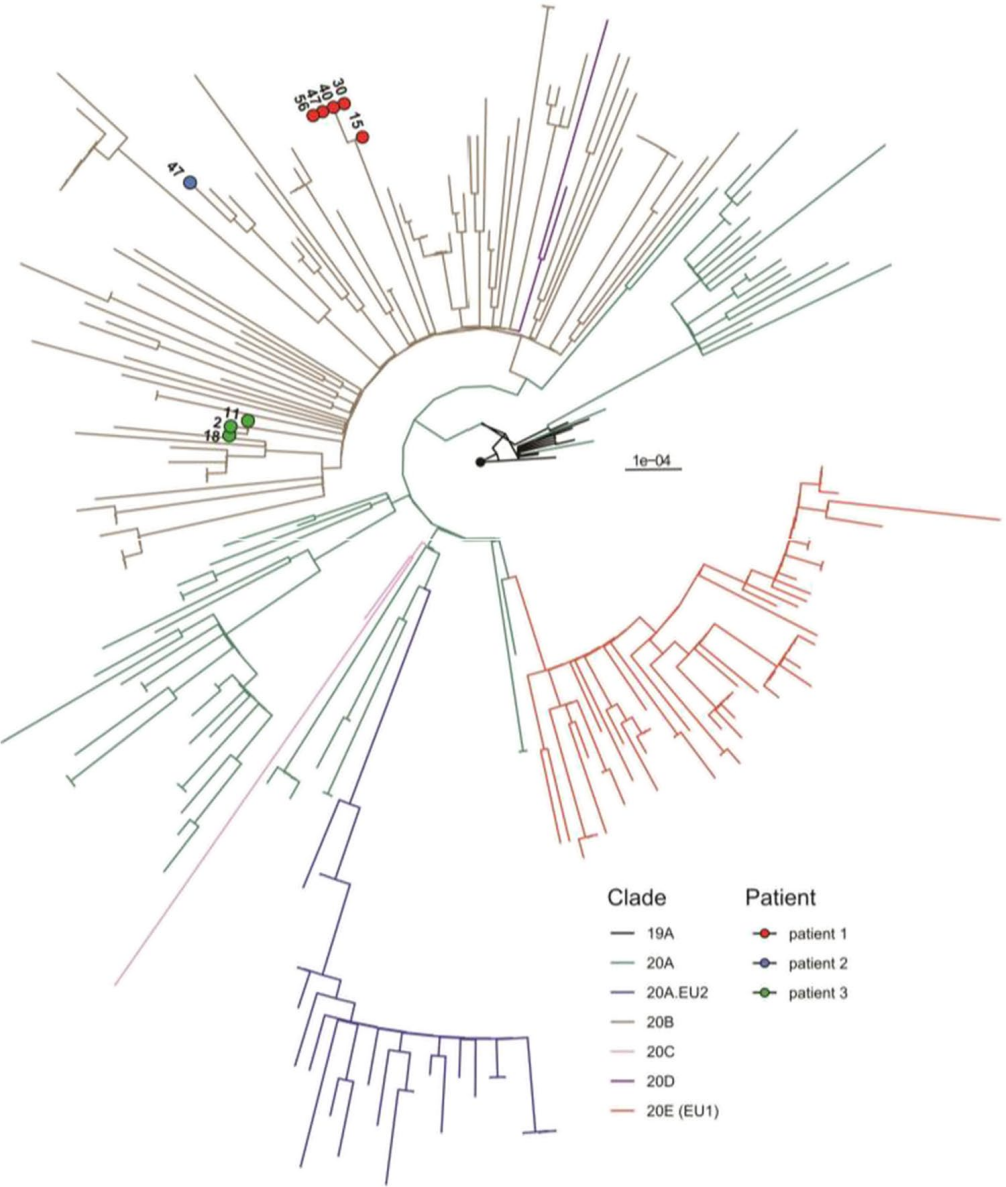
SARS-CoV-2 sequences obtained from oropharyngeal swabs of patients 1–3 were aligned to a set of randomly sampled SARS-CoV-2 genome sequences from Freiburg between October and November 2020 (deposited in the GISAID data bank (Fig. E1). The circularized maximum-likelihood phylogenetic tree was constructed with IQ-Tree (GTR + F + I) and rooted on the Wuhan-Hu-1 reference sequence (NC_045512), tree branches were colored according to their Nextclade classification. The scale represents nucleotide substitutions per site

**Fig. 3:**
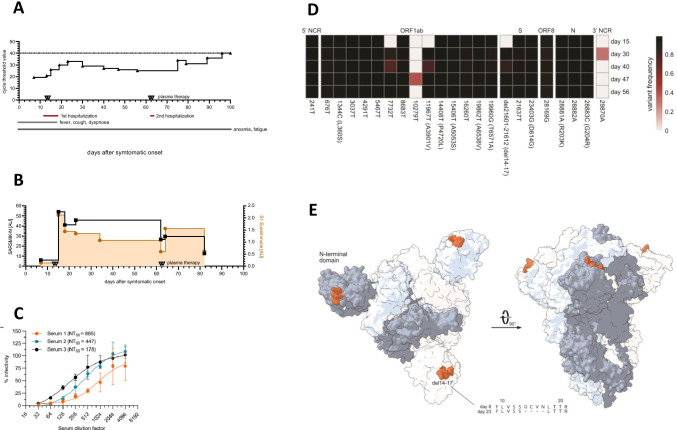
**A** Longitudinal cycle threshold values and **B** SARS-CoV-2 antibody titers in patient 1. AU = arbitrary units: SARS-CoV-2 S1 IgG Euroimmun ELISA (< 0.8 = negative); SARS-CoV-2 N IgG Mikrogen ELISA (< 23 = negative). **C** Neutralizing titer of convalescent plasma against a prototypic SARS-CoV-2 isolate. Neutralizing titers 50 (NT_50_) were calculated from the fitted curves. **D** Heatmap showing observed variant frequencies (> 10%) in the viral genome compared to Wuhan-Hu-1 reference sequence (NC_045512). Color intensity indicates the variant frequencies. **E** Spike structure is shown in surface representations (PDB accession number: 6vxx). Individual monomers are colored white, light blue and gray, respectively. The observed deletion at position 14–17 is marked red

To test for intra-host evolution of SARS-CoV-2, full-length SARS-CoV-2 genomes were analyzed from available oropharyngeal swabs of patients 1, 2, and 3. Phylogenetic analysis clustered all three patients into the clade 20B (Nextclade nomenclature) (Fig. 2) which was prevalent in Germany at the time of the initial infection (October to November). Longitudinal data after plasma treatment were only available for patients 1 and 3. Patient 3 acquired one mutation (T1890I in the ORF1ab) 5 days post plasma treatment but cleared the infection shortly thereafter (data not shown). For patient 1, we observed the emergence of a stable viral subpopulation 17 days after plasma treatment. Due to an increasing SARS-CoV-2 load (Fig. 3A), he was treated with a second course of convalescent plasma on day 62/63, resulting again in an immediate increase in nucleoprotein and spike specific antibodies (Fig. 3B) and finally viral clearance 13 days after the second treatment. Neutralization assays confirmed a high neutralization capacity of all convalescent plasma samples against a prototypic SARS-CoV-2 virus in vitro (Fig. 3C). The re-emerging virus carried a fixed in-frame deletion in viral spike gene (21,601–21,612) (Fig. 3D) resulting in the deletion of four surface exposed amino acids (del14-17) in the N-terminal domain (NTD) (Fig. 3E). This deletion is part of a N-terminal Domain supersite primarily targeted by neutralizing antibodies, and residues 14 to 17 have been shown to be epitope residues for multiple potently neutralizing NTD-directed antibodies [43, 44].Therefore, del14-17 might weaken antigen–antibody interactions or affect the conformation of this subdomain potentially conferring an antibody escape. The emergence of a potential escape mutation shortly after the first plasma treatment argues for a therapy-mediated immune pressure, which was absent before the immunological intervention.

## Discussion

In our case series of patients with primary antibody deficiency and COVID-19, convalescent plasma treatment was associated with a reduction in viral load and improvement of symptoms with the temporary appearance of SARS-CoV-2-specific antibodies. Importantly, this effect was not limited to treating high-risk patients early during infection, but also patients with persistent SARS-CoV-2 infection improved clinically, even when treated months after the first positive PCR result. This response is clearly distinct from the notion reported of immunocompetent patients [20–22] and is most likely due to the absence of an intrinsic humoral response in antibody-deficient patients a week after onset of symptoms when most immunocompetent patients with a severe disease have developed already a strong IgG response [45], suggesting a much wider window of therapeutic opportunity for antibody-based therapies in these patients [46]. This notion also confirms the effective role of neutralizing antibodies in the defense against persistent SARS-CoV2 infection suggested previously in other viral infections and mouse models propagated by Hangartner et al. [47]. In our study cohort, convalescent plasma treatment was safe and without serious side effects. Recently, a large cohort study in immunocompetent individuals raised concerns of possible side effects and different therapeutic efficacy of convalescent plasma products depending on distinct antibody profiles [22]. The convalescent plasma which we were able to test in our patient cohort had high antibody titers with detectable neutralizing capacity contributing to high therapeutic efficacy and low risk for side effects. We did not observe clinical signs of antibody-dependent enhancement (ADE) or enhanced respiratory disease (ERD) in any of our patients after plasma treatment [48] considered a risk especially in preparations with low titers of neutralizing antibodies. Another hypothesis is that antibody-deficient patients might tolerate convalescent plasma treatment better than immunocompetent patients. This idea is supported by case reports from patients with primary antibody deficiency and case series of patients with secondary antibody deficiency that tolerated convalescent plasma therapy well [23, 26, 27, 29, 46, 49]. However, underlying pathophysiological mechanisms remain unclear and further studies need to clarify this issue and the risk of ADE. Previous reports had suggested the risk of emerging escape variants secondary to plasma treatment [30–32]. Moreover, immunological considerations suspected that patients with persistent SARS-CoV-2 infection might enable viral evolution and represent a reservoir for newly emerging SARS-CoV-2 variants. Notably, we observed an increase in viral load, associated with the emergence of a possible antibody escape mutation in the SARS-CoV-2 genome after convalescent plasma treatment in one patient, indicating a selection pressure due to treatment. Interestingly, a persistent infection in patient 2 was not associated with the emergence of known escape variants 47 days after symptomatic onset. This is a case series and therefore only limited conclusions can be drawn about generalizability. Still, our data corroborate the idea of immunological, especially antibody driven selection pressure for the evolution of the specific variants in the RBD region that is lacking in immunocompromised hosts with persistent infection. Therefore, antibody-deficient patients with chronic infection might not generally represent a reservoir for evolution of new viral escape mutations; however, after treatment with convalescent plasma or monoclonal antibodies, regular follow-up with SARS-CoV-2 PCR becomes advisable in these patients. Reproducible increasing viral load after an initial decrease might be used as a hint for possible treatment failure and viral evolution.

Clear limitations of our case series are the small number of cases, combined therapies in some of the patients, and lack of close follow-up in the majority of patients. Therefore, it is not possible to draw definitive conclusions based on the reported cases. However, given the current lack of evidence on therapeutic management of patients with primary antibody deficiency and COVID-19, which according to our and other findings differs from immunocompetent patients, our observations are helpful information for physicians treating patients with primary antibody deficiency in this global pandemic. Where available, a standardized treatment with anti-SARS-CoV-2 monoclonal antibody cocktails should be considered as it showed promising results in early studies [50] and is not associated with the known adverse effects of plasma therapy including the potential transmission of anti-IFNalpha and IFNomega antibodies in plasma derived from convalescent patients [7]. However, the strength of convalescent plasma is the local access and cost-effectiveness of treatment, making it a valuable option in countries with limited access to monoclonal antibody therapies.

In summary, our data suggests sufficient safety and a beneficial effect of convalescent plasma therapy for patients with primary antibody deficiency due to severe COVID-19 or existing risk factors and even in persisting disease of more than 2 weeks. Plasma donors need to be carefully selected according to antibody profiles and treated patients need regular follow-up using SARS-CoV qPCR to identify a relapse in viral replication in time to avoid a time-dependent evolution of escape mutants. In the near future, prospective studies are needed to corroborate the findings of our case series.

## Supplementary Information

Below is the link to the electronic supplementary material.

Supplementary file1 (PDF 16 KB)

Supplementary file2 (PDF 393 KB)

## Data Availability

All necessary data and information are given in the manuscript. The sequence data are submitted to GISAID database and are publicly available (Fig E2).
