# Supplementary material for: Case Series: Convalescent Plasma Therapy for Patients with COVID-19 and Primary Antibody Deficiency
Source: J Clin Immunol. 2021 Dec 10;42(2):253–65. doi: 10.1007/s10875-021-01193-2 (PMC8664001; doi:10.1007/s10875-021-01193-2)
Supplement: Supplementary file 1 — Supplementary file1 (PDF 16 KB) [file 10875_2021_1193_MOESM1_ESM.pdf]

We gratefully acknowledge the following Authors from the Originating laboratories responsible for obtaining the specimens, as well as the Submitting laboratories where the genome data were generated and shared via GISAID, on which this research is based.

All Submitters of data may be contacted directly via www.gisaid.org

Authors are sorted alphabetically.

Fig E1

Accession ID	Originating Laboratory	Submitting Laboratory	Authors
EPI_ISL_1048308, EPI_ISL_1048315, EPI_ISL_1048328, EPI_ISL_1048329, EPI_ISL_1048333, EPI_ISL_1048334	FG17 - NRZ Influenzaviren	Robert Koch Institute, Influenza and respiratory viruses FG17 & Bioinformatics MF1, Berlin, Germany	R. Dürrwald, Stephan Fuchs, Stefan Kroeger, Marianne Wedde, Oliver Drechsel, Aleksandar Radonic, Rene Kmiecinski, Ralf Duerrwald, Thorsten Wolff
EPI_ISL_1092245, EPI_ISL_1092265, EPI_ISL_1092266, EPI_ISL_1092267, EPI_ISL_1092271, EPI_ISL_1092274, EPI_ISL_1092285, EPI_ISL_1092288, EPI_ISL_1092296, EPI_ISL_1092300	Protzer Lab	Protzer Lab, Laboratory for Functional Genome Analysis, Dept. Genomics, Gene Center of the LMU Munich	Ulrike Protzer, Dieter Hoffmann, Till Bunse, Eva C.Schulte, Elisabeth Esser, Stefan Krebs, Alexander Graf, Helmut Blum
EPI_ISL_1138603, EPI_ISL_1138604, EPI_ISL_1138607, EPI_ISL_1138621, EPI_ISL_1138623, EPI_ISL_1138625, EPI_ISL_1138628, EPI_ISL_1138634, EPI_ISL_1138635, EPI_ISL_1138642, EPI_ISL_1138651, EPI_ISL_1138653, EPI_ISL_1138654, EPI_ISL_1138655, EPI_ISL_1138656, EPI_ISL_1138664, EPI_ISL_1138671, EPI_ISL_1138672, EPI_ISL_1138673, EPI_ISL_1138677			
see above	Institut für Medizinische Virologie, Universitätsklinikum Frankfurt	Institut für Medizinische Virologie, Universitätsklinikum Frankfurt	Barbara Muehlemann et al
EPI_ISL_1140281, EPI_ISL_1140282, EPI_ISL_1140286, EPI_ISL_1140289, EPI_ISL_1140292, EPI_ISL_1140293, EPI_ISL_1140302, EPI_ISL_1140304, EPI_ISL_1140305, EPI_ISL_1140306, EPI_ISL_1140308, EPI_ISL_1140325, EPI_ISL_1140343, EPI_ISL_1140346, EPI_ISL_1140348, EPI_ISL_1140353, EPI_ISL_1140354, EPI_ISL_1140355, EPI_ISL_1140359, EPI_ISL_1140361, EPI_ISL_1140363, EPI_ISL_1140370, EPI_ISL_1140372, EPI_ISL_1140373, EPI_ISL_1140376, EPI_ISL_1140382, EPI_ISL_1140383, EPI_ISL_1140386, EPI_ISL_1140427			
see above	Labor ZOTZ|KLIMAS; MVZ Düsseldorf-Centrum	Robert Koch Institute	unknown
EPI_ISL_1141015	Labor Kneißler GmbH & Co. KG	Robert Koch Institute	unknown
EPI_ISL_1144913, EPI_ISL_1144915, EPI_ISL_1144918, EPI_ISL_1144919, EPI_ISL_1144922	Medizinische Hochschule Hannover Institut für Virologie	Robert Koch Institute	unknown
EPI_ISL_1160125, EPI_ISL_1160137, EPI_ISL_1160138, EPI_ISL_1160157, EPI_ISL_1160161	Center of Medical Microbiology, Virology, and Hospital Hygiene, University of Duesseldorf	Center of Medical Microbiology, Virology, and Hospital Hygiene, University of Duesseldorf	Maximilian Damagnez;Alexander Dilthey;Torsten Houwaart;Lisanna Hülse;Malte Kohns Vasconcelos;Nadine Lübke;Jessica Nicolai;Klaus Pfeffer;Daniel Strelow;Jörg Timm;Andreas Walker;Tobias Wienemann
EPI_ISL_1181340	Klinikum Bielefeld Mitte	Bielefeld University	David Brandt, Tobias Busche, Markus Haak, Jörn Kalinowski, Levin-Joe Klages, Alexander Sczyrba, Marina Simunovic, Svenja Vinke
EPI_ISL_1211991	Chemisches und Veterinäruntersuchungsamt Ostwestfalen-Lippe	Robert Koch Institute	unknown
EPI_ISL_1268859	Hannover Medical School, Institute of Virology	Hannover Medical School, Institute of Virology	Lars Steinbrück
EPI_ISL_1346717, EPI_ISL_1346730, EPI_ISL_1346746, EPI_ISL_1346747, EPI_ISL_1346752, EPI_ISL_1346754, EPI_ISL_1346755, EPI_ISL_1346757, EPI_ISL_1346760, EPI_ISL_1346762, EPI_ISL_1346769, EPI_ISL_1346943, EPI_ISL_1346945, EPI_ISL_1346946, EPI_ISL_1346947			
see above	Center of Medical Microbiology, Virology, and Hospital Hygiene, University of Duesseldorf	Center of Medical Microbiology, Virology, and Hospital Hygiene, University of Duesseldorf	Maximilian Damagnez;Alexander Dilthey;Torsten Houwaart;Lisanna Hülse;Malte Kohns Vasconcelos;Nadine Lübke;Jessica Nicolai;Klaus Pfeffer;Daniel Strelow;Jörg Timm;Andreas Walker;Tobias Wienemann
EPI_ISL_1355066	Universitätsklinikum Heidelberg	Robert Koch Institute	unknown
EPI_ISL_1373259, EPI_ISL_1373264, EPI_ISL_1373265, EPI_ISL_1373266, EPI_ISL_1373270, EPI_ISL_1373275, EPI_ISL_1373278, EPI_ISL_1373280, EPI_ISL_1373282, EPI_ISL_1373284, EPI_ISL_1373287, EPI_ISL_1373296, EPI_ISL_1373301, EPI_ISL_1373311, EPI_ISL_1373312			
see above	Protzer Lab	Protzer Lab, Institut für Medizinische Mikrobiologie und Hygiene, Gagneur Lab	Ulrike Protzer, Dieter Hoffmann, Till Bunse, Eva C.Schulte, Elisabeth Esser, Angel Angelov, Michael Sonnabend, Christina Engesser, Nicholas H.Smith, Julien Gagneur
EPI_ISL_1577827, EPI_ISL_1577829, EPI_ISL_1577832, EPI_ISL_1577837, EPI_ISL_1577839, EPI_ISL_1577840, EPI_ISL_1577844, EPI_ISL_1577849, EPI_ISL_1577863, EPI_ISL_1577876, EPI_ISL_1577878			
see above	Virology, Universitätsklinikum des Saarlandes	Epigenetics, Saarland University	Kathrin Kattler, Stefan Lohse, Sascha Tierling, Thorsten Pfuhl, Sigrun Smola, Jörn Walter
EPI_ISL_1588639	Institute of Virology, Medical Center, University of Freiburg, Freiburg, Germany	Institute of Virology, Clinial Virus Genomics, Medical Center, University of Freiburg, Freiburg, Germany	Jonas Fuchs, Lisa Kern, Lena Jaki, Sandra Reuter, Hajo Grundmann, Marcus Panning
EPI_ISL_1647998, EPI_ISL_1648006, EPI_ISL_1648008, EPI_ISL_1648010, EPI_ISL_1648012, EPI_ISL_1648025, EPI_ISL_1648033, EPI_ISL_1648036, EPI_ISL_1648043, EPI_ISL_1648044	Virology, Universitätsklinikum des Saarlandes	Epigenetics, Saarland University	Kathrin Kattler, Stefan Lohse, Sascha Tierling, Thorsten Pfuhl, Sigrun Smola, Jörn Walter
EPI_ISL_631307, EPI_ISL_631308, EPI_ISL_631310, EPI_ISL_631311, EPI_ISL_631313, EPI_ISL_631315, EPI_ISL_631318, EPI_ISL_631320, EPI_ISL_666623, EPI_ISL_666629	ZOTZ KLIMAS MVZ Düsseldorf-Centrum GbR ÜBAG für Labormedizin, Genetik, Zytologie, Pathologie	Center of Medical Microbiology, Virology, and Hospital Hygiene, University of Duesseldorf	Maximilian Damagnez, Alexander Dilthey, Ashley-Jane Duplessis, Patrick Finzer, Katrin Hoffmann, Torsten Houwaart, Lisanna Hülse, Malte Kohns Vasconcelos, Marek Korencak, Nadine Lübke, Jessica Nicolai, Klaus Pfeffer, Daniel Strelow, Jörg Timm, Andreas Walker, Tobias Wienemann, Rainer Zotz
EPI_ISL_707971, EPI_ISL_707993, EPI_ISL_708014, EPI_ISL_708015	Virology, Universitätsklinikum des Saarlandes	Epigenetics, Saarland University	Kathrin Kattler, Markus Vogelgesang, Stefan Lohse, Sascha Tierling, Sigrun Smola, Jörn Walter
EPI_ISL_718030, EPI_ISL_718031, EPI_ISL_718036, EPI_ISL_718062, EPI_ISL_718063, EPI_ISL_718068, EPI_ISL_718069, EPI_ISL_718072, EPI_ISL_718078, EPI_ISL_718081, EPI_ISL_718099			
see above	ZOTZ KLIMAS MVZ Düsseldorf-Centrum GbR ÜBAG für Labormedizin, Genetik, Zytologie, Pathologie	Center of Medical Microbiology, Virology, and Hospital Hygiene, University of Duesseldorf	Maximilian Damagnez, Alexander Dilthey, Ashley-Jane Duplessis, Patrick Finzer, Katrin Hoffmann, Torsten Houwaart, Lisanna Hülse, Malte Kohns Vasconcelos, Marek Korencak, Nadine Lübke, Jessica Nicolai, Klaus Pfeffer, Daniel Strelow, Jörg Timm, Andreas Walker, Tobias Wienemann, Rainer Zotz
EPI_ISL_729417, EPI_ISL_729424, EPI_ISL_729426, EPI_ISL_729427, EPI_ISL_729430, EPI_ISL_729441, EPI_ISL_729446, EPI_ISL_729467, EPI_ISL_729612, EPI_ISL_729618, EPI_ISL_729622, EPI_ISL_729624, EPI_ISL_729625, EPI_ISL_729627, EPI_ISL_729636, EPI_ISL_729639, EPI_ISL_729644, EPI_ISL_729646, EPI_ISL_729657, EPI_ISL_729659, EPI_ISL_729660, EPI_ISL_729661, EPI_ISL_729662, EPI_ISL_729663, EPI_ISL_729665, EPI_ISL_729666, EPI_ISL_729667, EPI_ISL_729675, EPI_ISL_729676, EPI_ISL_729679, EPI_ISL_729680, EPI_ISL_729681, EPI_ISL_729683, EPI_ISL_729686, EPI_ISL_729690, EPI_ISL_729691, EPI_ISL_729692, EPI_ISL_729693, EPI_ISL_729704, EPI_ISL_729729, EPI_ISL_729732			
see above	A. Krumbholz, Labor Dr. Krause und Kollegen MVZ GmbH, Kiel	Charité Universitätsmedizin Berlin, Institut für Virologie	Victor M Corman, Barbara Mühlemann, Jörn Beheim-Schwarzbach, Talitha Veith, Julia Schneider, Terry Jones, Christian Drosten
EPI_ISL_733503, EPI_ISL_733509, EPI_ISL_733511, EPI_ISL_733513, EPI_ISL_733515, EPI_ISL_733516, EPI_ISL_733517, EPI_ISL_733523	ZOTZ KLIMAS MVZ Düsseldorf-Centrum GbR ÜBAG für Labormedizin, Genetik, Zytologie, Pathologie	Center of Medical Microbiology, Virology, and Hospital Hygiene, University of Duesseldorf	Maximilian Damagnez, Alexander Dilthey, Ashley-Jane Duplessis, Patrick Finzer, Katrin Hoffmann, Torsten Houwaart, Lisanna Hülse, Malte Kohns Vasconcelos, Marek Korencak, Nadine Lübke, Jessica Nicolai, Klaus Pfeffer, Daniel Strelow, Jörg Timm, Andreas Walker, Tobias Wienemann, Rainer Zotz

EPI_ISL_753784, EPI_ISL_753789, EPI_ISL_753794, EPI_ISL_753795, EPI_ISL_753802, EPI_ISL_753803, EPI_ISL_753804, EPI_ISL_753815, EPI_ISL_753816, EPI_ISL_753818, EPI_ISL_753859, EPI_ISL_753865, EPI_ISL_753867, EPI_ISL_753868, EPI_ISL_753870, EPI_ISL_753872, EPI_ISL_753879, EPI_ISL_753881, EPI_ISL_753883, EPI_ISL_753892, EPI_ISL_753893, EPI_ISL_753903, EPI_ISL_753912, EPI_ISL_753916, EPI_ISL_754188, EPI_ISL_754192, EPI_ISL_754193

see above	Charité Universitätsmedizin Berlin, Institut für Virologie/Labor Berlin	Charité Universitätsmedizin Berlin, Institut für Virologie	Victor M Corman, Jörn Beheim-Schwarzbach, Barbara Mühlemann, Julia Schneider, Talitha Veith, Terry Jones, Christian Drosten
EPI_ISL_763047	Unit 17: Influenza & Other Respiratory Viruses, German National Influenza Center	Project group Epidemiology of Highly Pathogenic Microorganisms, Robert Koch-Institute	Ariane Düx, Andreas Sachse, Grit Schubert, Sébastien Calvignac-Spencer, Fabian Leendertz, Thorsten Wolff, Ralf Dürrwald, Djin-Ye Oh, Marianne Wedde
EPI_ISL_770718	ZOTZ KLIMAS MVZ Düsseldorf-Centrum GbR ÜBAG für Labormedizin, Genetik, Zytologie, Pathologie	Center of Medical Microbiology, Virology, and Hospital Hygiene, University of Duesseldorf	Maximilian Damagnez, Alexander Dilthey, Ashley-Jane Duplessis, Patrick Finzer, Katrin Hoffmann, Torsten Houwaart, Lisanna Hülse, Malte Kohns Vasconcelos, Marek Korencak, Nadine Lübke, Jessica Nicolai, Klaus Pfeffer, Daniel Strelow, Jörg Timm, Andreas Walker, Tobias Wienemann, Rainer Zotz
EPI_ISL_779935	Center of Medical Microbiology, Virology, and Hospital Hygiene, University of Duesseldorf	Center of Medical Microbiology, Virology, and Hospital Hygiene, University of Duesseldorf	Maximilian Damagnez, Alexander Dilthey, Ashley-Jane Duplessis, Torsten Houwaart, Lisanna Hülse, Malte Kohns Vasconcelos, Nadine Lübke, Jessica Nicolai, Klaus Pfeffer, Daniel Strelow, Teresa Tamayo, Jörg Timm, Andreas Walker, Tobias Wienemann
EPI_ISL_796059, EPI_ISL_796060, EPI_ISL_796061	Institute of Virology, University of Cologne	Institute of Virology, University of Cologne	Saleta Sierra, Gibran Rubio, Zevanya Tessalonica, Dominik Aschenmeier, Eva Heger, Elena Knops, Rolf Kaiser, Maximilian Damagnez, Andreas Walker, Jörg Timm, Alexander Dilthey, Martin Däumer, Alex Thielen
EPI_ISL_830735	University Hospital Basel, Clinical Virology	University Hospital Basel, Clinical Bacteriology	Tim Roloff, Madlen Stange, Helena MB Seth-Smith, Alfredo Mari, Karoline Leuzinger, Julia Bielicki, Manuel Battegay, Hans Hirsch, Adrian Egli
EPI_ISL_852599, EPI_ISL_852600	Max von Pettenkofer Institute, Virology, National Reference Center for Retroviruses, LMU München	Laboratory for Functional Genome Analysis, Dept. Genomics, Gene Center of the LMU Munich	Max Muenchhoff, Stefan Krebs, Alexander Graf, Oliver Keppler, Helmut Blum
EPI_ISL_852633, EPI_ISL_852634, EPI_ISL_852637, EPI_ISL_852638, EPI_ISL_852645, EPI_ISL_852649, EPI_ISL_852650	Institute of Virology, Medical Center, University of Freiburg, Freiburg, Germany	Institute of Virology, Clinial Virus Genomics, Medical Center, University of Freiburg, Freiburg, Germany	Jonas Fuchs, Lisa Kern, Sandra Reuter, Hajo Grundmann, Marcus Panning
EPI_ISL_853923	Pharmgenetix GmbH	Bergthaler laboratory, CeMM Research Center for Molecular Medicine of the Austrian Academy of Sciences	Lukas Endler, Alexandra Popa, Benedikt Agerer, Jakob-Wendelin Genger, Alexander Lercher, Anna Schedl, Thomas Penz, Michael Schuster, Jan Laine, Martin Senekowitsch, Christoph Bock, Andreas Bergthaler
