# Supplementary material for: Case Series: Convalescent Plasma Therapy for Patients with COVID-19 and Primary Antibody Deficiency
Source: J Clin Immunol. 2021 Dec 10;42(2):253–65. doi: 10.1007/s10875-021-01193-2 (PMC8664001; doi:10.1007/s10875-021-01193-2)
Supplement: Supplementary file 2 — Supplementary file2 (PDF 393 KB) [file 10875_2021_1193_MOESM2_ESM.pdf]

Fig E2

Patient 1		
**Days after symtomatic onset**	**days after first plasma treatment**	**GISAID accession number**
15	2	EPI_ISL_1195401
30	17	EPI_ISL_1195404
40	27	EPI_ISL_1195405
47	34	EPI_ISL_1195407
56	43	EPI_ISL_1195408

Patient 2		
**Days after symtomatic onset**	**days after plasma treatment**	**GISAID accession number**
47	-1	EPI_ISL_1357684

Patient 3		
**Days after symtomatic onset**	**days after first plasma treatment**	**GISAID accession number**
2	-11	EPI_ISL_1195400
11	-2	EPI_ISL_1195402
18	5	EPI_ISL_1195403
